# Time from Exposure to Diagnosis among Quarantined Close Contacts of SARS-CoV-2 Omicron Variant Index Case-Patients, South Korea

**DOI:** 10.3201/eid2804.220153

**Published:** 2022-04

**Authors:** Hye Ryeon Lee, Young June Choe, Eun Jung Jang, Jia Kim, Ji Joo Lee, Hye Young Lee, Hanul Park, Sang Eun Lee, Moonsu Kim, Seonggon Kim, Hanna Yoo, Ju-Hyung Lee, Hyun Jeong Ahn, Mi-Young Go, Won Ick Kim, Bu Sim Lee, Hwa-Pyeong Ko, Jeonghee Yu, Eun-Young Kim, Hyoseon Jeong, Jae-Hwa Chung, Jin Su Song, Jihee Lee, Mi Young Kim, Young-Joon Park

**Affiliations:** Korea Disease Control and Prevention Agency, Cheongju, South Korea (H.R. Lee, E.J. Jang, J. Kim, J.J. Lee, H.Y. Lee, H. Park, S.E. Lee, Y.-J. Park);; Korea University Anam Hospital, Seoul, South Korea (Y.J. Choe);; Incheon Metropolitan City, Incheon, South Korea (M. Kim, S. Kim, H. Yoo);; Jeollabuk-do Center for Infectious Disease Control and Prevention, Jeonju, South Korea (J.-H. Lee);; Jeollabuk-do Government, Jeonju (H.J. Ahn, M.-Y. Go);; Jeollanam-do Government, Muan, South Korea (W.I. Kim, B.S. Lee); Gwangju Metropolitan Government, Gwangju, South Korea (H.-P. Ko);; Honam Reginal Center for Disease Control and Prevention, Gwangju (J. Yu, E.-Y. Kim, H. Jeong, J.-H. Chung);; Capital Regional Center for Disease Control and Prevention, Seoul (J.S. Song, J. Lee, M.Y. Kim)

**Keywords:** COVID-19, 2019 novel coronavirus disease, coronavirus disease, severe acute respiratory syndrome coronavirus 2, SARS-CoV-2, viruses, respiratory infections, zoonoses, Omicron, variant, VOC

## Abstract

To determine optimal quarantine duration, we evaluated time from exposure to diagnosis for 107 close contacts of severe acute respiratory syndrome coronavirus 2 Omicron variant case-patients. Average time from exposure to diagnosis was 3.7 days; 70% of diagnoses were made on day 5 and 99.1% by day 10, suggesting 10-day quarantine**.**

Since its identification in November 2021, the severe acute respiratory syndrome coronavirus 2 (SARS-CoV-2) Omicron variant of concern has rapidly spread across the globe (*1*). In South Korea, the first case-patient infected with the Omicron variant was identified on November 24, 2021, among inbound international travelers (*2*). In response, all close contacts of Omicron case-patients were required to quarantine for 14 days, regardless of vaccination and symptom status (*3*). Prolonged quarantine of close contacts of persons infected with newly emerging pathogens may pose a substantial burden on society. To determine optimal quarantine duration, we quantified time from exposure to diagnosis among close contacts of SARS-CoV-2 Omicron index case-patients. The study was conducted as a legally mandated public health investigation under the authority of government activity.

The study population consisted of close contacts of 2 clusters of SARS-CoV-2 Omicron case-patients detected on November 24 and 25, 2021. All quarantined close contacts were actively monitored by public health officers and were tested with reverse transcription PCR on days 1, 9, and 13 (*4*). We retrieved demographic information from the epidemiologic investigation form. Coronavirus disease (COVID-19) vaccination status was verified through documentation. Presence of fever or worsening or onset of symptoms was assessed daily, and persons were tested when deemed necessary. We calculated the time from exposure to the index case-patient and diagnosis of SARS-CoV-2 for close contacts in days (mean, median, interquartile range). We used the inverse Kaplan-Meier curve to visualize the percentage of diagnoses in a given interval. 

We identified 107 close contacts of SARS-CoV-2 Omicron index case-patients, and the average time from exposure to diagnosis (± SD) was 3.7 (± 2.6) days (Table; Figure). We [Fig F1]calculated the cumulative cases by time from exposure to diagnosis for total cases according to age group, according to vaccination status, and according to symptom status. Among all case-patients, diagnoses were made for 50% on day 3, and for 70% on day 5 (Figure, panel A). By day 10, almost all (106 [99.1%]) diagnoses had been made; 1 diagnosis was made on day 13 for an unvaccinated child with a previous negative test result. For all age groups (Figure, panel B), diagnoses were made for 50% on day 3; among those >20 years of age, for 70% on day 4; and among those <20 years of age, on day 5 (p = 0.051). Among close contacts who were symptomatic on the day of encounter, diagnoses were made for 50% on day 3 and 70% on day 5. Among asymptomatic close contacts, diagnoses were made for 50% on day 5 and 70% on day 8 (p = 0.001) (Figure, panel D).

**Table:** Time from close contact exposure to index case-patients infected with severe acute respiratory syndrome coronavirus 2 Omicron variant of concern to diagnosis of infection, South Korea*

Variable	Close contacts, no. (%)	Time from exposure to diagnosis, d	
		Average (± SD)	Median IQR (range)
All close contacts	107	3.7 (± 2.6)	3.0 (2–5)
Age group, y			
<20	37 (34.6)	4.4 ± (3.0)	4.0 (3–6)
20–59	64 (59.8)	3.3 (± 2.5)	3.0 (1–5)
>60	6 (5.6)	3.7 (± 2.0)	4.0 (1.8–5.3)
Sex			
F	56 (52.3)	3.5 (± 2.5)	3.0 (1.3–5)
M	51 (47.7)	4.0 (± 2.8)	3.0 (2–5)
Symptom status			
Symptomatic	85 (79.4)	3.3 (± 2.1)	3.0 (2–5)
Asymptomatic	22 (20.6)	5.2 (± 3.8)	5.0 (2–9)
Vaccination status			
Not vaccinated	58 (54.7)	4.0 (±2.7)	3.0 (2–5)
Partially vaccinated	6 (5.7)	3.0 (± 2.1)	2.5 (1–5.3)
Fully vaccinated	42 (39.6)	3.4 (± 2.7)	3.0 (1–5)

*IQR, interquartile range.

**Figure F1:**
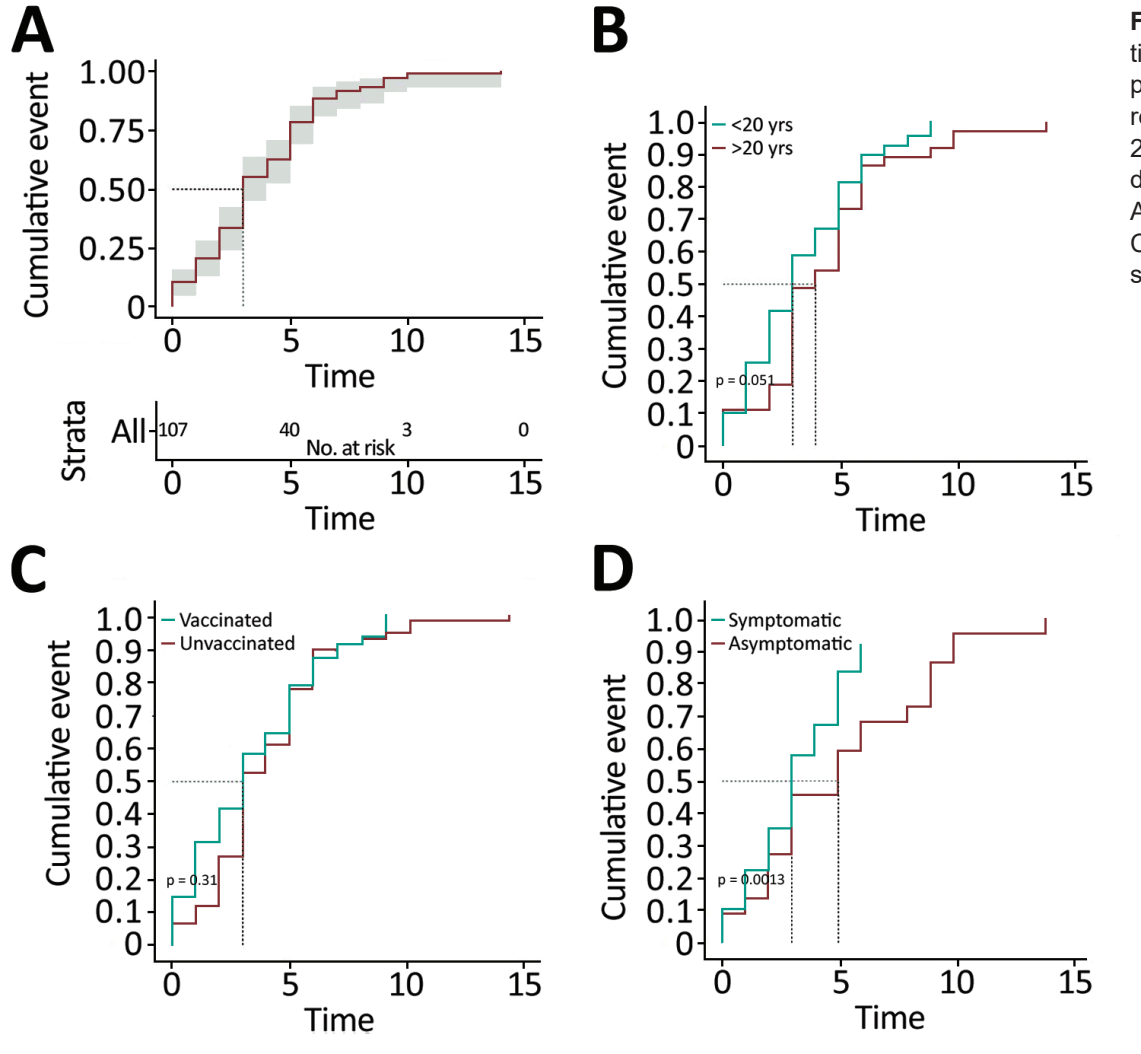
Cumulative events by time from exposure to index case-patients infected with severe acute respiratory syndrome coronavirus 2 Omicron variant of concern to diagnosis of infection, South Korea. A) Total cases; B) by age group; C) by vaccination status; and D) by symptom status.

We found that most (99.1%) diagnoses for the close contacts of the Omicron index case-patients were made within 10 days of quarantine; mean interval varied according to symptom status. Supported by this finding, the quarantine duration in South Korea was shortened from 14 to 10 days for all case-patients and even shorter (to 7 days) if quarantine facilities were at capacity because of a surge of cases (*3*). 

Among the limitations of our study, individual public health officers decided to test on days 2–8 and 10–12; therefore, the chance of being tested on any day may have differed for each close contact. Nonetheless, early testing may have been driven by the presence of symptoms and influenced by an individual close contact’s desire to get tested, which may have affected the decision of public health officers. Moreover, because tests were not performed after day 13, we do not have information on long incubation periods. Given that the study population was from 2 distinctive clusters, incubation periods may differ according to characteristics of illness in the index case-patients, which may affect study results. Despite these limitations, were able to quantify the time from exposure to diagnosis and estimate the optimal duration of quarantine for persons exposed to Omicron.

Implementing quarantine early in a pandemic is crucial for slowing the spread of a novel pathogen (*5*). Previous studies have suggested that the incubation period for Omicron could be shorter than that for the SARS-CoV-2 Delta variant (J. Chen, unpub. data, https://arxiv.org/abs/2112.01318). Estimating the duration of infectiousness is more challenging than measuring incubation periods; one study that measured viral load of Omicron suggested that viral load had diminished by days 10–13, which is in line with our findings (*6*).

To mitigate spread of highly contagious pathogens, the most effective public health measures are isolation and quarantine; however, these measures inevitably lead to personal and socioeconomic costs, necessitating evidence-based guidance from policy makers. A 10-day quarantine period may encompass most persons exposed to Omicron; however, quarantine duration may become shorter after balancing societal cost with public health benefit.
